# Public involvement in the design and conduct of clinical trials: a narrative review of case examples

**DOI:** 10.1186/1745-6215-12-S1-A82

**Published:** 2011-12-13

**Authors:** Jonathan Boote, Wendy Baird, Anthea Sutton

**Affiliations:** 1 NIHR Research Design Service for Yorkshire and the Humber, South Yorkshire, S1 4DA, UK; 2 School of Health and Related Research, University of Sheffield, South Yorkshire, S1 4DA, UK

## Background

Public involvement is health research policy in the UK and internationally. There is a need to establish a robust evidence base on the impact of public involvement on research processes and outcomes.

## Aim

To review case examples of public involvement in the design and conduct of clinical trials, to synthesise the contributions of the public, as well as the identified tensions and facilitating strategies.

## Method

Systematic literature search and narrative review.

## Findings

Nine papers were identified, covering the following topics: breast-feeding, antiretroviral and nutrition interventions [1]; paediatric resuscitation [2]; exercise and cognitive behavioural therapy [3]; hormone replacement therapy and breast cancer [4]; stroke [5,6]; chronic suppurative otitis media [7]; Paget's disease [8]; and shared decision-making in patient consultations [9]. Six papers reported on public involvement at the trial design stage, while three reported on public involvement at the design and conduct stages of clinical trials. It was found that the public contributed at the consultation, collaboration and publicly-led levels of involvement. Four main public contributions to trial design were identified: review of consent procedures and patient information sheets; suggestion of additional trial outcomes; review of trial data collection procedures; and recommendations on the timing and location of trial follow-up data collection. Two main contributions that the public made to the conduct of trials were identified: scrutiny of the conduct of the trial through membership of the Trial Steering Committee; and delivering the trial protocol after completing relevant training. Four main tensions were identified with regard to involving the public in trial design and conduct: tensions between stakeholder groups when designing trials; public understanding of trial methodology; the added time, complexity and cost of public involvement; and the representativeness of the public involved. Four main facilitating strategies were identified with regard to involving the public in trial design and conduct: cultural sensitivity; clear explanation of trial methodology; independent facilitation of trial design planning meetings; and adequate funding for public involvement.

## Limitations

Papers on public involvement in the design and conduct of clinical trials may have been overlooked due to the difficulty of searching for, and identifying, papers in this area. Only publications published in English were searched for and the review focused on evidence contained in peer-reviewed journal articles only.

## Conclusions

The issues raised in this review should assist researchers in developing and conducting clinical trials with the involvement of the public.
